# Spatial–Temporal Distribution Variation of Ground-Level Ozone in China’s Pearl River Delta Metropolitan Region

**DOI:** 10.3390/ijerph18030872

**Published:** 2021-01-20

**Authors:** An Zhang, Jinhuang Lin, Wenhui Chen, Mingshui Lin, Chengcheng Lei

**Affiliations:** 1 State Key Laboratory of Resources and Environmental Information System, Institute of Geographical Sciences and Natural Resources Research, Chinese Academy of Sciences, Beijing 100101, China; zhangan@igsnrr.ac.cn (A.Z.); leicc.16b@igsnrr.ac.cn (C.L.); 2 School of Geography and Ocean Science, Nanjing University, Nanjing 210023, China; 3 College of Geographical Science, Fujian Normal University, Fuzhou 350007, China; chenwenhui@fjnu.edu.cn; 4 College of Tourism, Fujian Normal University, Fuzhou 350117, China

**Keywords:** ground-level ozone, spatial–temporal kriging, spatial–temporal distribution, spatial clustering, centers of gravity

## Abstract

Long-term exposure to ozone pollution will cause severe threats to residents’ physical and mental health. Ground-level ozone is the most severe air pollutant in China’s Pearl River Delta Metropolitan Region (PRD). It is of great significance to accurately reveal the spatial–temporal distribution characteristics of ozone pollution exposure patterns. We used the daily maximum 8-h ozone concentration data from PRD’s 55 air quality monitoring stations in 2015 as input data. We used six models of STK and ordinary kriging (OK) for the simulation of ozone concentration. Then we chose a better ozone pollution prediction model to reveal the ozone exposure characteristics of the PRD in 2015. The results show that the Bilonick model (BM) model had the highest simulation precision for ozone in the six models for spatial–temporal kriging (STK) interpolation, and the STK model’s simulation prediction results are significantly better than the OK model. The annual average ozone concentrations in the PRD during 2015 showed a high spatial variation in the north and east and low in the south and west. Ozone concentrations were relatively high in summer and autumn and low in winter and spring. The center of gravity of ozone concentrations tended to migrate to the north and west before moving to the south and then finally migrating to the east. The ozone’s spatial autocorrelation was significant and showed a significant positive correlation, mainly showing high-high clustering and low-low clustering. The type of clustering undergoes temporal migration and conversion over the four seasons, with spatial autocorrelation during winter the most significant.

## 1. Introduction

Ground-level ozone is an essential trace component of the atmosphere and is mainly synthesized when NOx and VOC in natural and anthropogenic emissions undergo photochemical reactions upon exposure to ultraviolet radiation [1,2]. High concentrations of ozone can severely affect human health, damaging the central nervous system and pulmonary, visual, and thyroid functions [3]. Long-term inhalation of high ozone concentrations can increase the incidence and severity of asthma and lung diseases and damage the cardiovascular system [4,5,6]. China sets the guideline value for ozone levels at 100 µg/m^3^ (0.053 ppm) for the daily maximum 8-h mean in China’s Ambient air quality standards(GB 3095—2012). WHO (World Health Organization) also sets the same value as the AQG (Air Quality Guideline). According to the 2016 China Environmental Bulletin released by the Ministry of Ecology and Environment [7], ozone has become an air pollutant in the Beijing–Tianjin–Hebei and Yangtze River Delta regions that is second only to particulate matter (PM_2.5_). In the Pearl River Delta, ozone has replaced PM_2.5_ to become the most important air pollutant. With increasing urbanization in the Pearl River Delta Metropolitan Region (PRD), pollution from industrial, vehicular traffic, and urban sources have increased the ozone concentrations, which persist at relatively high levels [8]. Therefore, ground-level ozone pollution is an environmental issue in the PRD, and the ozone pollution exposure assessment has become an urgent need in PRD.

Many studies are dealing with the spatial–temporal characteristics of air pollution [9,10]. Most of these studies focus on particulate matter pollutants visible to the naked eye from the research object. These include PM_2.5_ and PM_10_. However, there is less research on the pollution resulting from ozone. Ozone pollution cannot be seen with the naked eye but is usually more harmful, and high concentrations of ozone can cause many adverse effects on human health, agricultural production, and ecosystems [11]. From the research methods, current studies on the spatial–temporal ozone characteristics mainly use land-use regression models, atmospheric dispersion models, and interpolation models [12]. However, due to the limitations of temporal precision of auxiliary data in land-use regression models, these models are unsuitable for simulation and prediction studies of small-scale regions [13,14,15]. The simulation process for atmospheric dispersion models requires the input of multiple complex environment variables simultaneously. Therefore, data acquisition with a high spatial resolution is difficult and costly for large areas [16,17]. Researchers usually use interpolation models for spatial interpolation based on the actual measured data from monitoring stations and do not sufficiently consider temporal interpolation. Therefore, they cannot accurately reveal the spatial–temporal patterns of ozone [18,19]. From the research content, the current research on the ozone’s spatial–temporal characteristics mainly analyzes the spatial distribution, temporal changes, and regional differentiation characteristics of ozone. There is little analysis of the regional migration characteristics and spatial clustering of ozone [2,7].

The spatial–temporal kriging (STK) model is a kind of interpolation simulation in the space–time dimension, which can not only make up for the interpolation in the time dimension of the traditional spatial interpolation method but also quickly realize the large-scale simulation prediction of ozone. Therefore, we used the daily maximum 8-h ozone concentration from 55 national ozone monitoring sites in the PRD in 2015 as input data. We used six models of STK and ordinary kriging (OK) for the simulation of ozone concentration. After that, we selected a better ozone concentration estimation model based on the cross-validation method. In the end, we analyzed the variation’s characteristics of spatial–temporal distribution, spatial clustering, and migration patterns of centers of gravity for the PRD ozone to reveal the spatial–temporal distribution patterns of ozone pollution in the PRD in 2015. This study will improve the evaluation of ozone health risks and better provide a scientific basis for the formulation of targeted recommendations for areas with a high risk of ozone exposure, promoting ozone reduction strategies and reducing the risk of ozone pollution exposure.

## 2. Study Site, Data, and Methods

### 2.1. Overview of the Study Area

**Figure 1:**
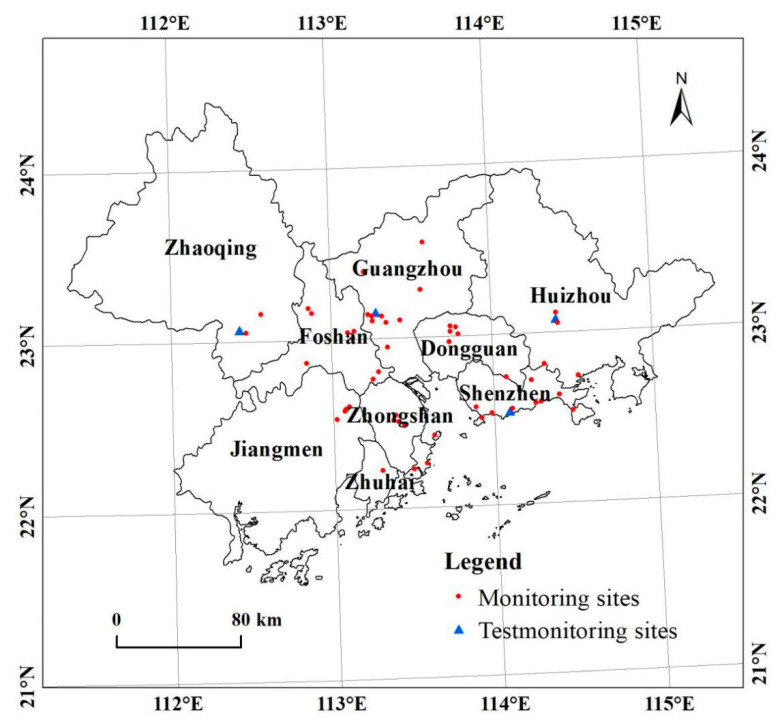
The geographical location of the study area.

The PRD is located at 111.35°–116.73° E, 20.69°–24.40° N, at central-southern Guangdong Province, China, and is separated from Southeast Asia by the sea (Figure 1). There are nine prefecture-level cities in the PRD, including Guangzhou, Shenzhen, Huizhou, Dongguan, Zhaoqing, Foshan, Zhongshan, Zhuhai, and Jiangmen. The total urban area is 42,200 km^2,^ and this urban area has the most economic activity and the highest level of urbanization of the three major urban areas in China. This region has a flat terrain consisting mainly of plains and hills, with an elevation lower than 200 m. The region has a South Asian tropic monsoon climate with high temperatures and rainfall in the summer and mild, drier winters. Air monitoring data show that ozone pollution in this region is dangerous, and ozone is often the leading air pollutant. Ozone poses a severe threat to the health of the residents.

### 2.2. Study Methods

#### 2.2.1. Data and Methods

We used the daily maximum 8-h ozone concentration from 55 national monitoring sites in the PRD in 2015. The data source was the National real-time city’s air quality release platform in the China National Environmental Monitoring Center (http://106.37.208.233:20035/). We used Wuhu, Liyuan, Xiapuhengjiangsanluzi, and Muganzi stations from the east, south, west, and north directions to validate ozone predictions. We used the OK and STK models for interpolation at a 2 × 2 km resolution based on the daily maximum 8-h ozone concentrations over 365 d at the 51 monitoring sites. We calculated the root-mean-square error (RMSE) of the two interpolation methods to compare the OK and STK models’ interpolation results. We used the most superior interpolation method for an in-depth analysis of the spatial–temporal evolution, regional differentiation, spatial clustering, and migration patterns for centers of gravity based on the daily, monthly, seasonal, and yearly average ozone concentrations in 2015. We point out the 2015 variation characteristics for the spatial–temporal distribution of ozone concentrations in the PRD with the interpolation results. We generated all the maps in the Albers map projection by using ArcGIS 10.1 software.

#### 2.2.2. Construction of Predictive Models

##### Ordinary Kriging (OK) Model

The OK model uses the variation function of geographical factors and the raw data’s structural characteristics for unbiased, linear optimal interpolation estimation of spatial variables. This method overcomes the difficulty of analyzing interpolation errors and carries out a point by theoretical point estimation of the error. Besides, this method does not produce the boundary effects seen in regression analysis, and it is a spatial interpolation method with unbiased estimation [20].

##### Spatial–Temporal Kriging (STK) Model

There are two kinds of STK models, including separable and nonseparable models. There three separable models, including the Bilonick model (BM), Dagum model (DM), and Ma model (MM). While, there are three nonseparable models, including Cressie-Huang model 1 (CH1), Cressie-Huang model 2 (CH2), and Gneiting model (GM) [21,22,23,24,25,26]. The partial submodels have corresponding submodels at the spatial and temporal dimensions. Among them, there are specific differences in the parameters of different STK modes. However, the main factors that significantly impact the models’ fitting effect are the parameters in the time dimension, space dimension, and spatial–temporal dimensions, such as spatial–temporal variation function spatial–temporal nugget effect, and spatial distance of spatial–temporal variables, etc. Details of the models have been described previously described [18,19,27,28,29].

#### 2.2.3. Model Error Analysis

We computed the mean relative error (MRE) to help judge the STK model fit’s goodness. We used the root-mean-square error (RMSE) to compare the OK and STK models’ predictions of ozone concentrations in the PRD. The RMSE assumes that the test stations’ ozone concentrations are unknown before using the known data from surrounding stations for prediction to compare the error magnitude of the two methods.

#### 2.2.4. Calculation of Daily Average Concentration

We selected means of 8-h averages over a given time for each station and regional mean values based on spatial statistics to compare the effects of the two different concentration estimation methods on estimating ozone concentrations in the PRD. The calculation formula is as follows.

(1) $$Z(X)=\frac{1}{n}\sum_{i=1}^{n}Z(X_{i})$$

(2) $$Z(X^{\prime })=\frac{1}{n^{\prime }}\sum_{i^{\prime }=1}^{n^{\prime }}Z(X_{i^{\prime }})$$

Here, *Z(X)* and *Z*(X′) are the daily average concentrations based on station statistics and spatial statistics, respectively, *n* and *n*′ are the number of raster for the number of monitoring sites, and after interpolation, *Z*(*X_i_*)** and *Z*(*X*_*i*′_)** are the values of the *i*th monitoring site and the *i*′th raster.

#### 2.2.5. Spatial Autocorrelation

To describe the spatial autocorrelation of the ozone concentrations distribution, we used the global Moran’s I and local Moran’s I. Based on estimation results for ozone concentrations in the PRD, and we used a 2 × 2 km grid as an evaluation unit to calculate the spatial autocorrelation for ozone. Additionally, we used the LISA (local indicators of spatial association) cluster map to analyze its spatial clustering characteristics.

The calculation formula for the global Moran’s I [30] is as follows.

(3) $$I_{Global}=\frac{\sum_{i=1}^{n}\sum_{j=1}^{n}w_{ij}\left(x_{i}-\bar{x}\right)\left(x_{j}-\bar{x}\right)}{\sum_{i=1}^{n}\sum_{j=1}^{n}w_{ij}\sum_{i=1}^{n}{\left(x_{i}-\bar{x}\right)}^{2}}$$

The calculation equation for the local Moran’s I [31] is as follows.

(4) $$I_{L\mathrm{ocal}}=\frac{\left(X_{i}-\bar{X}\right)}{S^{2}}\sum_{j}W_{\mathrm{ij}}\left(X_{j}-\bar{X}\right),$$

where $I_{Global}$ represents the global Moran’s I index and $I_{L\mathrm{ocal}}$ represents the local Moran’s I index, *X_i_* and *X_j_* represent the mean sensitivity index in the *i*th and *j*th evaluation unit, respectively. $\overset{-}{X}$ represents the mean sensitivity index of the entire evaluation unit, *W_ij_* represents the spatial weight matrix, and *S* represents the sum of various elements in the spatial weight matrix.

#### 2.2.6. Distribution of Centers of Gravity of Ozone

The center of gravity calculation uses the geometric center model to output the new point feature class. This study used the ozone concentrations in a 2 × 2 km grid as a basis and employed the regional weighted core model to calculate the centers of gravity of ozone concentrations in the PRD during the 12 months of 2015. We extracted the migration trajectory for these centers of gravity, allowing analysis of the migration variation characteristics of ozone concentrations between PRD regions. The extraction equation for centers of gravity is as follows.

(5) $${\bar{X}}_{w}=\frac{\sum_{i=1}^{n}w_{i}x_{i}}{\sum_{i=1}^{n}w_{i}}\;{\bar{Y}}_{w}=\frac{\sum_{i=1}^{n}w_{i}y_{i}}{\sum_{i=1}^{n}w_{i}},$$

where *x_i_* and *y_i_* are the coordinates of element *i*, *n* is the total number of elements, and *w_i_* is the weight of element *i*.

## 3. Results and Analysis

### 3.1. Comparison of STK Models

Before carrying out STK, we set the temporal resolution, time step, spatial resolution, and space step to 1 d, 10 d, 1 km, and 15 km, respectively. We computed the mean relative error (MRE) of the three separable models (BM, DM, and MM) and three nonseparable models (CH1, CH2, and GM) for model fitting error. From the mean relative error (MRE) of the models, we found that BM, DM, MM, GM, NH2 and NH1 are 0.0401, 0.0491, 0.0519, 0.0532, 0.0624, 0.0629, respectively. Referring to the results of previous studies on the STK model [18,19], generally, when the MRE is less than 0.1, the model fitting effect is better. The lower the MRE of the model, the better its effect and then provides higher accuracy. The BM model had the smallest MRE. Table 1 shows the Bilonick model’s parameters and errors for STK interpolation of ozone concentrations in the PRD.

### 3.2. Comparison of OK and STK Models

We compared the STK and OK models for the spatial–temporal interpolation of ozone. With regards to the STK interpolation method, the BM model had the highest fitting accuracy. We selected the BM model for spatial–temporal interpolation of daily maximum 8-h ozone concentration data. The spatial–temporal ozone simulation’s temporal resolution predicted result is 1 d, and the spatial resolution is 2 × 2 km. For the OK model, we used identical monitoring site data for simulation and prediction analysis of daily ozone concentrations. We used the monitoring ground-truth data and prediction values from the four test stations to cross-validate the STK and OK models. Table 2 showed the RMSEs of the OK and STK models were 16.51 and 14.81 µg/m^3^, respectively. The result shows that the STK model’s simulation and prediction results for ozone concentrations in the PRD were better than the OK model.

### 3.3. Annual Average Ozone Concentrations and Migration of Centers of Gravity

**Figure 2:**
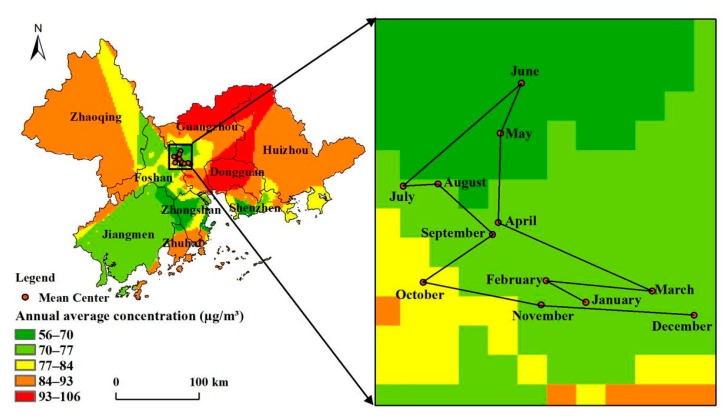
Spatial distribution of annual average ozone concentration and average center variation in the Pearl River Delta Metropolitan Region.

We calculated the annual average ozone concentration of the PRD region in 2015 by using the STK model’s interpolation results. The annual average ozone concentrations showed significant spatial heterogeneity and an overall trend of high concentrations in the north and east and low concentrations in the south and west (Figure 2). The regions with the highest annual average ozone concentrations were mainly concentrated in mid-western Dongguan, northwestern Huizhou, and northeastern Guangzhou, indicating high ozone pollution concentrations for long periods.

From the migration trajectory of ozone concentration centers of gravity from January to December (Figure 2), the centers of gravity showed a pattern of initially migrating to the north and west, before migrating to the south, and finally migrating to the east. The centers of ozone concentrations’ gravity moved northward from January to June. Then, the centers moved westward from June to July. Finally, the centers moved eastward from October to December. These data show that ozone in the PRD undergoes seasonal changes in migration and dispersion.

**Figure 3:**
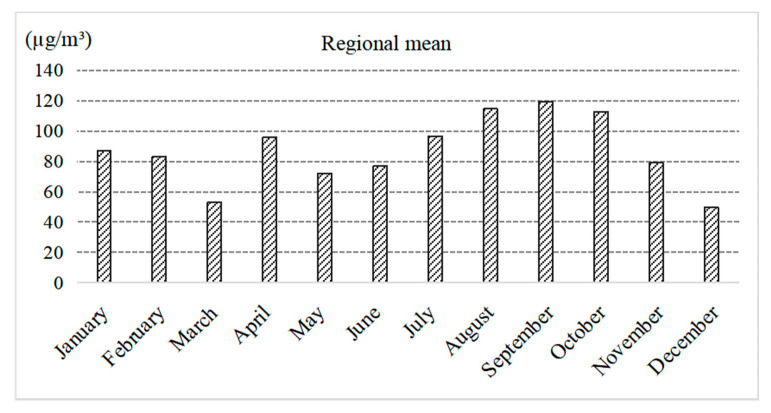
Monthly means of ozone concentration in the Pearl River Delta Metropolitan Region.

The variation curve of average ozone concentrations from January to December showed that the PRD’s monthly average ozone concentration initially decreased, then fluctuated before decreasing again (Figure 3). The lowest ozone concentration appeared in March, while the highest ozone concentration appeared in September. The concentrations in August and October were also relatively high. This variation was mainly due to changes in the direction of the prevailing winds and seasonal changes in solar radiation intensity.

### 3.4. Seasonal Ozone Changes and Regional Differentiation

**Figure 4:**
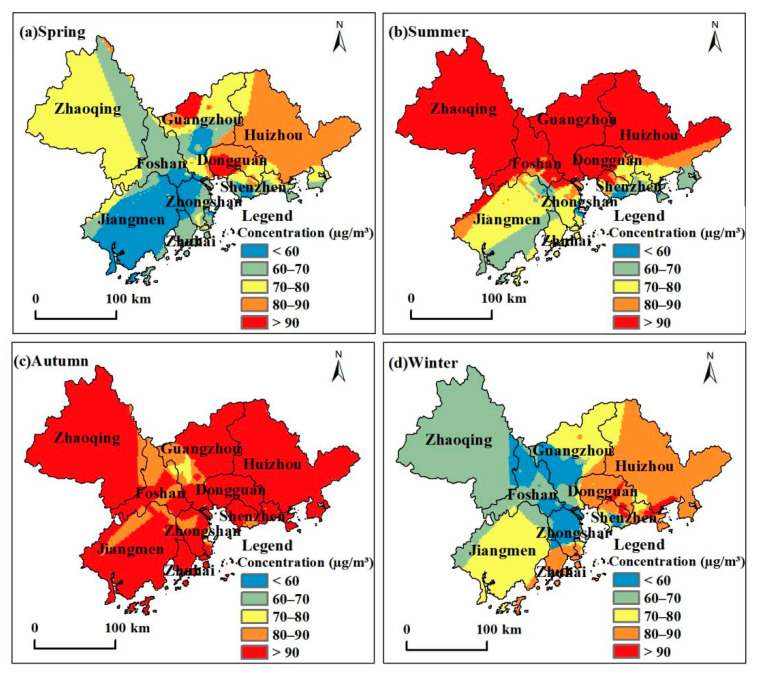
Spatial distribution of ozone concentration during spring, summer, autumn, and winter in the Pearl River Delta Metropolitan Region.

We used the criteria for delineating the four seasons of ozone concentrations in the Pearl River Delta established in a previous study [11] to divide the ozone concentrations in the PRD in 2015 into spring (March to May), summer (June to August), autumn (September to November), and winter (December to February). We calculated the spatial distribution characteristics of the cumulative mean values. The ozone concentrations in the PRD tended to be high in summer and autumn and low in winter and spring (Figure 4). The seasonal average ozone concentrations were the highest in autumn. The location of the PRD region is similar to Hong Kong SAR, China. A study [32] in Hong Kong SARfound that the seasonal variation of ozone concentration in this region is mainly affected by the solar radiation intensity and the wind direction variation of monsoon. The intense solar radiation in summer and autumn leads to a significant increase in ozone concentration in the atmosphere. 

The seasonal average ozone concentrations in the nine prefecture-level cities and the entire region were used for regional statistics to illustrate the regional differentiation and seasonal variation characteristics of ozone concentrations (Table 3). Ozone concentrations in the PRD in 2015 showed clear regional differentiation because of the difference between coastal and inland. The regions with the highest spring ozone concentrations were mainly concentrated in inland cities such as Dongguan and Huizhou, while the spring concentration was the lowest in coastal cities such as Jiangmen. In summer, ozone concentrations were highest in inland cities such as Guangzhou, Zhaoqing, and Dongguan, while coastal cities such as Shenzhen and Zhuhai had the lowest concentrations. In autumn, all the region cities had high ozone concentrations. In winter, Dongguan and coastal cities such as Huizhou, Shenzhen, and Zhuhai had the highest ozone concentrations. Overall, Dongguan, Huizhou, and Zhuhai cities had a higher annual average ozone concentration while Jiangmen and Foshan had lower annual average ozone concentrations.

**Figure 5:**
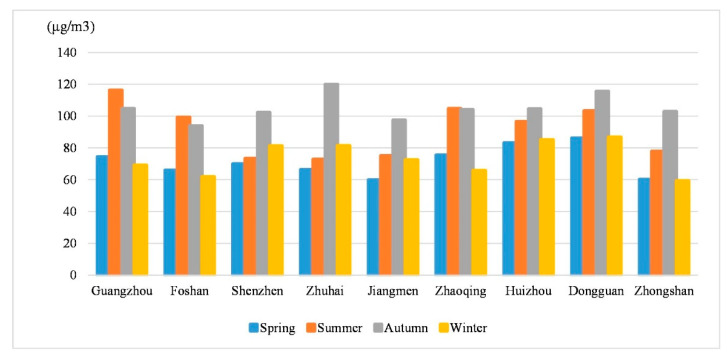
Seasonal variation characteristics of ozone concentrations in nine prefecture-level cities in the Pearl River Delta Metropolitan Region.

The distribution curve of seasonal ozone concentrations is in Figure 5. We divided the PRD’s nine prefecture-level cities into three major categories with seasonal variation characteristics. In the first category (using Guangzhou and Foshan as representatives), the seasonal variation characteristics for ozone concentrations were winter < spring < autumn < summer. In the second category (using Shenzhen, Zhuhai, and Jiangmen as representatives), the seasonal variation characteristics for ozone concentrations were spring < summer < winter < autumn. In the third category (using Zhaoqing, Huizhou, Dongguan, and Zhongshan as representatives), the seasonal variation characteristics for ozone concentrations were winter < spring < summer < autumn.

### 3.5. Spatial Clustering Characteristics of Ozone in the Four Seasons

We used ozone concentrations in a 2 × 2 km grid as a basis for the calculation of global spatial autocorrelation and local spatial autocorrelation for ozone concentrations in the PRD (Table 4). In 2015, the global Moran’s I for ozone concentrations in the PRD were all greater than 0, autumn < summer < spring < winter, and the Z-score was significantly higher than 1.96. Therefore, ozone distribution in the PRD showed significant spatial autocorrelation, and the correlation was significantly positive. Besides, in winter, ozone concentrations showed the most significant spatial clustering, while autumn had the weakest spatial clustering. The regional variation of ozone concentrations in winter is considerable, while the change is less in autumn.

**Figure 6:**
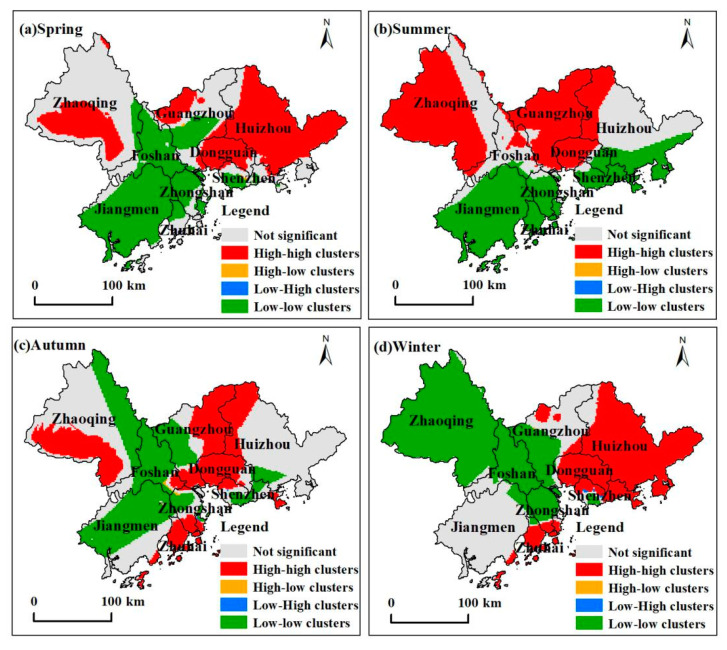
Local spatial autocorrelation of ozone concentration during spring, summer, autumn, and winter in the Pearl River Delta Metropolitan Region.

Figure 6 shows the local spatial autocorrelation during 2015 for ozone in the PRD. The spatial cluster characteristics in the four seasons mainly consisted of high-high clusters (H-H) and low-low clusters (L-L) while high-low (H-L) and low-high (L-H) clusters distributed sparsely. Additionally, the spatial cluster characteristics showed significant spatial heterogeneity. During seasonal changes, the types of clusters also undergo spatial migration and conversion. From spring to summer, high-high clusters converted from large clusters in east and west to large clusters in west and north. The western H-H clusters expanded while the H-H clusters in the east migrated to the north. Some L-L clusters in the center moved to the southeast. Spatially, L-L clusters showed an overall northward migration trend, and some insignificant northern areas converted to L-L clusters. From autumn to winter, H-H clusters distributed in the east. The long-term H-H clusters in the west were converted to L-L clusters while L-L clusters continued their northward migration and expansion.

## 4. Discussion

### 4.1. Seasonal Change of Ozone in Different Regions

This study is based on the ozone concentration monitoring site in 2015 to study the characteristics of the spatial–temporal changes of ozone in the PRD and found that ozone pollution in the PRD has significant seasonal changes and showed a trend of high concentrations in summer and autumn and lower concentrations in winter and spring. However, many countries in the world have paid attention to the harmfulness of ozone pollution, and its seasonal characteristics, such as Europe and the United States have successively deployed long-term observation sites around urban areas, and forming a relatively mature observation network, and organized many large-scale field observation projects to study ozone [33,34,35]. Many studies mainly focus on ozone concentration monitoring, precursor emissions, and meteorological elements affecting ozone production. Studies have generally found that ozone pollution has specific seasonal changes. Besides, based on revealing the characteristics of the spatial pattern of regional ozone concentration and its main influencing factors, a targeted strategy to effectively reduce the hazards of ozone pollution in the region is proposed, which provides a scientific basis for regional ozone control.

### 4.2. Limitations and Outlook

Based on the STK model and ozone concentration monitoring data, this study attempts to reveal the spatial–temporal evolution characteristics of ozone concentration in the PRD in 2015. Compared with the traditional OK spatial interpolation model, the STK model considers both spatial and temporal interpolation and has a higher accuracy of the simulation and prediction of ozone concentration. The research also enriches the theories and methods of air pollution exposure assessment to a certain extent. However, the research still has some shortcomings. For example, although we considered the time dimension in the STK model, the interpolation result is greatly affected by the spatial distribution and density of monitoring stations. When the stations are fewer and unevenly distributed, the accuracy of the interpolation result is less stable. Besides, we did not discuss many more of the influencing factors in this paper. Thus, we want to improve the ozone concentration’s interpolation result and analyze influencing factors in the future.

## 5. Conclusions

We used the daily maximum 8-h ozone concentration data from the PRD in 2015 as a basis for the analysis of the spatial–temporal characteristics of ozone, including spatial–temporal evolution, regional differentiation, spatial clustering, and migration patterns for centers of gravity. We reached the following significant conclusions:

(1) We compared the STK separable models (BM, DM, and MM) and the nonseparable (NH1, NH2, and GM) models. The MRE for the BM model was the smallest, and it produced the best result as the STK fitting model for ozone concentrations. The STK model’s simulation and prediction results were significantly better than the OK model and more accurately revealed the spatial–temporal variation of ozone concentrations in the PRD.

(2) The annual average ozone concentrations for the PRD in 2015 were relatively high in the north and east and low in the south and west. The lowest ozone concentration appeared in March, while the highest appeared in September. The centers of gravity of ozone concentrations first migrated to the north and west, migrated to the south, and finally migrated to the east.

(3) The ozone concentrations for the PRD in 2015 showed a trend of high concentrations in summer and autumn and lower concentrations in winter and spring. We divided the PRD’s cities into three major categories with seasonal variation characteristics. Regional differentiation of maximum and minimum ozone concentrations in the four seasons was evident. However, the annual average ozone concentrations for Dongguan, Huizhou, and Zhuhai cities were relatively high, while Jiangmen and Foshan cities were relatively low.

(4) The ozone concentrations for the PRD in 2015 show significant positive spatial autocorrelation. Seasonally, spatial autocorrelation was the strongest in winter and the weakest in autumn. The spatial clustering characteristics mainly consisted of high-high and low-low clusters, which undergo spatial migration and conversion with seasonal changes, which can better reflect the spatial clustering characteristics of ozone in different seasons and provide a scientific basis for the formulation of targeted recommendations for areas with a high risk of ozone exposure.

## Figures and Tables

**Table 1:** Parameters and errors of the Bilonick model for daily average ozone concentration.

Model	Parameters and Errors							
	MRE	*C*_0_	*C_s_*	*C_t_*	*a_t_*	*C_st_*	*a_st_*	*a*
BM	0.0401	0.0142	3.9873 × 10^−8^	0.0626	6.8434	0.0031	7005.2376	1325.1108

Where BM is Bilonick model, MRE is mean relative error, *C*_0_ is the spatial–temporal nugget effect, *C_s_* is the spatial dimension coefficient, *h_s_* is the spatial distance of space–time variables, *C_t_* is the time dimension arch height, *a_t_* is the time dimension variation, *a_st_* is the spatial–temporal dimension variation, *a* is the spatial–temporal geometric anisotropy ratio.

**Table 2:** Comparison of error analysis between Ordinary Kriging (OK) model and Spatial-Temporal Kriging (STK) model.

OK Model		STK Model	
MAE (µg/m^3^)	RMSE (µg/m^3^)	MAE (µg/m^3^)	RMSE (µg/m^3^)
10.90	16.51	10.76	14.81

**Table 3:** Seasonal average ozone levels in various cities in urban areas of the Pearl River Delta.

City	Spring (µg/m^3^)	Summer (µg/m^3^)	Autumn (µg/m^3^)	Winter (µg/m^3^)
Guangzhou	74.42	116.29	104.77	69.10
Shenzhen	70.04	73.37	102.31	81.29
Zhuhai	66.35	72.89	119.93	81.47
Foshan	66.01	99.27	93.93	61.91
Jiangmen	59.94	75.09	97.52	72.47
Zhaoqing	75.55	104.81	104.13	65.83
Huizhou	83.17	96.66	104.51	85.14
Dongguan	86.22	103.34	115.57	86.82
Zhongshan	60.26	77.90	102.87	59.28
Entire region	73.20	96.29	103.23	72.87

**Table 4:** Local spatial autocorrelation of seasonal ozone concentration in the Pearl River Delta Metropolitan Region.

Season	Moran’s I	Z Score
Spring	0.8728	1078.873
Summer	0.8553	1057.248
Autumn	0.7584	937.548
Winter	0.8815	1089.748

## Data Availability

Data available on request from the authors.
